# Microbiological Sulfide Removal—From Microorganism Isolation to Treatment of Industrial Effluent

**DOI:** 10.3390/microorganisms9030611

**Published:** 2021-03-16

**Authors:** Zhendong Yang, Zhenghua Liu, Aleksandra Sklodowska, Marcin Musialowski, Tomasz Bajda, Huaqun Yin, Lukasz Drewniak

**Affiliations:** 1 Institute of Microbiology, Faculty of Biology, University of Warsaw, Miecznikowa 1, 02-096 Warsaw, Poland; zyang@biol.uw.edu.pl (Z.Y.); asklodowska@biol.uw.edu.pl (A.S.); marcinmusialowski@student.uw.edu.pl (M.M.); 2 School of Minerals Processing and Bioengineering, Central South University, No. 932 Lushan South Road, Changsha 410083, China; lzh2019@csu.edu.cn (Z.L.); yinhuaqun_cs@sina.com (H.Y.); 3 Faculty of Geology, Geophysics and Environmental Protection, AGH University of Science and Technology in Krakow, A. Mickiewicza 30, 30-059 Krakow, Poland; bajda@agh.edu.pl

**Keywords:** sulfide-oxidizing bacteria, sulfur production, sulfide depletion, sulfate reduction

## Abstract

Management of excessive aqueous sulfide is one of the most significant challenges of treating effluent after biological sulfate reduction for metal recovery from hydrometallurgical leachate. The main objective of this study was to characterize and verify the effectiveness of a sulfide-oxidizing bacterial (SOB) consortium isolated from post-mining wastes for sulfide removal from industrial leachate through elemental sulfur production. The isolated SOB has a complete sulfur-oxidizing metabolic system encoded by *sox* genes and is dominated by the *Arcobacter* genus. XRD analysis confirmed the presence of elemental sulfur in the collected sediment during cultivation of the SOB in synthetic medium under controlled physicochemical conditions. The growth yield after three days of cultivation reached ~2.34 g_protein_/mol_sulfid_, while approximately 84% of sulfide was transformed into elemental sulfur after 5 days of incubation. Verification of isolated SOB on the industrial effluent confirmed that it can be used for effective sulfide concentration reduction (~100% reduced from the initial 75.3 mg/L), but for complete leachate treatment (acceptable for discharged limits), bioaugmentation with other bacteria is required to ensure adequate reduction of chemical oxygen demand (COD).

## 1. Introduction

Hydrometallurgical effluents containing sulfate and heavy metals are produced widely from streamlines of, for example, electronics, mining, and electroplating industries [1]. Metal sulfide precipitation followed by biogenic sulfide generation of sulfate-reducing bacteria was frequently applied to metal recovery from industrial effluents instead of chemical approaches, such as ion exchange, adsorption, and electrolysis, etc. [2,3,4] because of its low cost and high efficiency [5]. However, the high concentration of sulfide significantly restricts the activity of sulfate-reducing bacteria [6]. In addition, sulfide is a highly toxic substance concerned with odor and corrosion and can be released into the air through volatilization as H_2_S gas, posing a risk to human health. Methods like adding biochar were proposed to decrease its toxicity to the microbial community in the sulfate-reducing system [7]. However, free sulfide cannot be completely contained because a lack of sulfide also notably limits metal removal by precipitation [8]. On the other hand, 1 mg/L of sulfide is already considered corrosive [9], with an odor threshold at 0.18 mg/L [10]. Therefore, it is necessary to treat the remaining sulfide after metal sulfide precipitation before such water enters the environment.

Removing sulfide often refers to its oxidation either to elemental sulfur or sulfate [11,12]. Sulfate is produced when the complete oxidation of sulfide is realized. This is in contrast to the production of biogenic sulfides, and it is less attractive to treat excessive sulfide after sulfate reduction. While the aim of a circular economy focuses on sustainable production from waste, transforming sulfide to elemental sulfur by partial oxidation is attracting more attention. Elemental sulfur shows considerable application potential in soil amendment [13]. Chemical oxidants such as KMnO_4_ and H_2_O_2_ have been successfully used to produce sulfur from sulfide [14]. However, the biological oxidation of sulfides to sulfur with sulfide-oxidizing bacteria (SOB), which are also known as sulfur compound oxidizing bacteria, is more commonly used to reduce the cost of this process [15,16,17]. 

Many studies reported the application of SOB to produce sulfur by oxidizing sulfide, of which chemolithoautotrophs are of the greatest interest [17,18]. With the presence of limited oxygen supply, these bacteria utilize inorganic reduced sulfur, especially sulfide, as the electron donors and energy source for metabolic activities, and CO_2_ as the carbon source. Studies with a special focus on removing sulfide in the gas phase were conducted. A good example is the THIOPAQ^®^ technology that oxidizes sulfide producing sulfur in a synthetic medium after washing off H_2_S gas adsorbed in a column [19]. The SOB work effectively in a controlled system, where sulfur is formed as the terminal product instead of sulfate. The optimal parameters, e.g., pH, oxygen level, and temperature, for chemolithoautotrophic-producing sulfur were well-elucidated by Krayzelova et al. (2015) [18]. However, actual industrial wastewater presents a more difficult and complex environment for the survival of SOB, which has to compete with other bacteria. Not only sulfide but other contents need to be depleted, such as high COD (chemical oxygen demand). Changing the physicochemical conditions, in this case, for spontaneous removal of all contaminants becomes relatively difficult.

This work aims to evaluate an SOB consortium isolated from post-mining wastes and explore new strategies for biological sulfide depletion and sulfur production in terms of aqueous industrial effluent. We focused on the metabolic functions and physiological characteristics of the microbial consortium. Particular emphasis was placed on its use in the treatment of water loaded with sulfides and high COD after biological reduction of sulfates. Specifically, we sought to address the following questions: (i) What are the sulfide oxidizers in the consortium; (ii) how does the SOB consortium produce sulfur under optimal conditions in the synthetic medium, and (iii) how can the SOB consortium be used for treating industrial effluent?

## 2. Materials and Methods

### 2.1. Microbial Consortium for Sulfide Oxidation

Samples were taken from post-mining wastes deposited in a dump located near a uranium mine in Radoniow, Poland. The characteristics of these wastes were described by Rewerski et al. (2014) [20]. Serial passaging of the SOB consortium was carried out on a modified Beijerinck medium [21] (MB medium): Na_2_S_2_O_3_ (10 g/L), KH_2_PO_4_ (4 g/L), K_2_HPO_4_ (4 g/L), NH_4_Cl (1.6 g/L), MgSO_4_·7H_2_O 0.8 g/L. In MB medium, thiosulfate was used (instead of sulfide), which is often applied for the isolation of obligate chemolithoautotrophic SOB [22]. It is easily soluble and non-toxic, while the external sulfur in S_2_O_3_^2−^ ion has an oxidation state of −2 as sulfide ion. For the isolation process of SOB consortium, the production of S[0] is not the targeting aspect. Further, standard potential (in volts) for the oxidation of thiosulfate to sulfate is lower than sulfide to sulfate [23]. Thus, employing thiosulfate as the substrate of electron donor for SOB enables complete oxidation to sulfate S[6+], which is easier for process control: (i) thiosulfate causes no volatilization; (ii) no controlled oxygen supply is needed. After a series of 10 passages on the MB medium, the consortium was subjected to detailed metagenomic analysis dedicated to illustrating the sulfur microbial structure and metabolic pathways.

For the first passage, 10 g of sample was added to a 500 mL Erlenmeyer flask filled with 200 mL of MB medium. The flask was shaken at room temperature (120 rpm) for three days. Afterwards, 100 mL of cell suspension was collected and centrifuged. The cell pellet was then resuspended in 200 mL of fresh MB medium poured into a new Erlenmeyer flask for the next passage. The passage process was repeated 10 times, then 100 mL of culture from the last passage was cultivated in a mixing reactor (20 rpm) filled with MB medium for a long-run. The cell culture from the reactor was supplied as the inoculum source of SOB consortium for the following procedure: 100 mL of cell culture was collected and centrifuged. The cell pellet was then resuspended in 0.85% saline solution for further use (cell count at ~10^6^). The inoculum was characterized by 16S rDNA approaches described in Section 2.4.

### 2.2. Experimental Set-Up

#### 2.2.1. Na_2_S Bio-Oxidation and Sulfur Production

**Figure 1:**
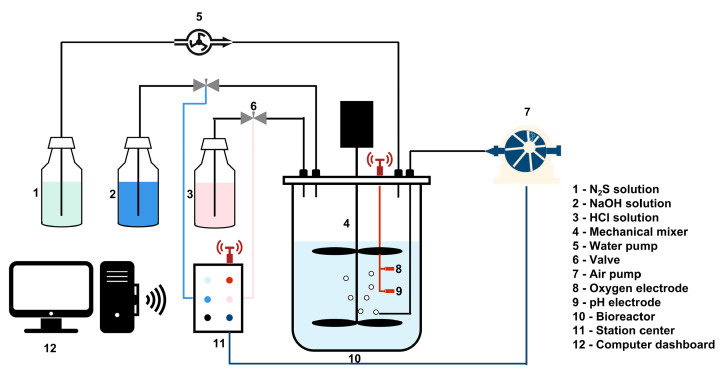
Scheme of the experimental setup for biological sulfide oxidation.

A semi-continuous reactor system was employed for the Na_2_S oxidation. The configuration of the system was presented in Figure 1. Within the following work, sulfide (including S^2−^, HS^−^, and dissolved H_2_S) was referred to as S[2−], while elemental sulfur was referred to as S[0]. Sulfur with +6 state in sulfate was referred to as S[6+]. The saturation of the solution with oxygen was kept at 1%. The Na_2_S stock solution was slowly fed to the reactor by a pump with an even speed (47.1 mL/day). To prepare the Na_2_S stock solution, Na_2_S·9H_2_O was dissolved in 1% NaOH, where the S[2−] concentration was set up to 4 g/L. In order to reduce the sulfide losses and H_2_S volatilization caused by pH increase and accumulation of S[2−] in the culture, the system was equipped with an automatic pH balanced circuit, dosing 1% HCl and 1% NaOH to adjust the pH steadily at 8.35. Two variants were set up for the experiment and were carried out in a separate 5-day run, respectively: (1) bio-oxidation: 900 mL of modified MB medium (without Na_2_S_2_O_3_) was filled in the reactor, then 100 mL of inoculum was inoculated in the reactor; (2) chemical control: one liter of modified MB medium (without Na_2_S_2_O_3_) was filled in the reactor. During the operation of the system, 10 mL of sample was collected each day from the reactor for the determination of SO_4_^2−^, S[2−] and total protein concentration, as well as the cell count in the culture. After 5 days, all culture from reactor was centrifuged, while separated sediment was then collected and dried in the air at room temperature for one week. The dried sediment was weighed and sent to XRD analysis.

#### 2.2.2. Two-Stage Bio-Oxidation of Industrial Effluent

The industrial effluent is collected from an up-flow anaerobic sludge bed (UASB) reactor for the treatment of acidic leachate (pH = 0.45) from the recycling of alkaline batteries [24]. In the UASB reactor, sulfate reducing bacteria produces sulfide which further precipitated with metals for metal recovery. However, part of the excessive sulfide (H_2_S) went into the effluent requiring additional treatment. A two-stage bio-oxidation strategy was adapted to remove chemical oxygen demand (COD), Volatile fatty acids (VFAs), and sulfide (S[2−]) from the effluent, partially using the system described above (the pH balance and Na_2_S dosing systems were disabled): (i) Stage I: The SOB consortium was employed to reduce the S^2−^ to elemental sulfur under micro-aerobic condition. (ii) Stage II: a commercial BioRemOil consortium (http://rdls.pl/, accessed on 1 December 2020), containing 10 strains representing the following genera: *Achromobacter*, *Alacligenes*, *Bacillus*, *Pseudomonas*, *Ochrobactrum*), were employed to remove VFAs and COD in the effluent. To start the run, one liter of the effluent was put into the reactor following by the addition of 100 mL of SOB inoculum. The saturation of the solution with oxygen was kept at 1% from day 0 to 5. At the day 6, 100 mL of BioRemOil preparation (cell count at ~10^6^) was added to the culture, whereas the oxygen saturation was lifted to 100% and kept at this level until the end of the experiment. A control variant was carried out by only changing the saturation of the solution with oxygen at day 5 (from 1% to 100%) without the inoculation. Ten milliliters of sample was collected each day for the determination of COD, VFA, S[2−] and SO_4_^2−^ concentration.

### 2.3. Analytical Methods

#### 2.3.1. Chemical Analysis

The determination of COD, VFA, SO_4_^2−^ concentration was performed by the photometer tube test (Nanocolor^@^ PF-12, MACHEREY-NAGEL GmbH & Co. KG, Düren, Germany). Specifically: Kits 0–29 was used for the determination of COD concentration; Kits 0–50 was used for the determination of VFAs; Kits 0–62 and 0–87 were used for the determination of SO_4_^2−^ concentration (S[6+] concentration equals SO_4_^2−^ concentration times 32 divided by 98). Elemental sulfur content (S[0]) was identified by the method described by Bartlett and Skoog (1954) [25]. The parameters setting and testing procedures followed the protocol of each kit provided by the manufacturer. The concentration of S[2−] was determined colorimetrically, followed by the incorporation of S[2−] into methylene blue with the presence of N,N-dimethyl-p-phenylenediamine. The testing procedure was as follows: In an empty Nanocolor glass test tube, (i) add 0.5 mL DPD solution (0.02 M *N*,*N*-dimethyl-p-phenylenediamine sulfate in 7.2 N HCl); (ii) add 0.5 mL Ferric solution (0.03 M ferric chloride in 1.2 N HCl); (iii) add 1 mL 5% (*w*/*v*) zinc acetate solution; (iv) add 1 mL sample and vortex vigorously; (v) deposit the tube into dark for 20 min and measure the absorbance at 620 nm by Nanocolor^@^ PF-12. The standard curve presenting the correlation of absorbance and S[2−] concentration was prepared by testing diluted Na_2_S solutions (in 1% NaOH) with known concentrations.

#### 2.3.2. Solid Phase Analysis

The powder X-ray diffraction Debye–Scherrer–Hull (XRD) method was adapted to identify the sulfur in the sediment after Na_2_S bio-oxidation. The dry sediment was finely ground and sieved to particles with a diameter less than 0.2 mm. The Rigaku SmartLab X-ray diffractometer was used to record the diffractograms of the sieved solid samples. The analytic parameters were set as follows: CuKα radiation, a graphite reflection monochromator, tube voltage 45 kV, tube current 200, and step scan mode (step size = 0.05°2θ, per 1 s). To identify the phase pattern of the solid samples, the interplanar distances were applied based on data provided in the catalogue of the International Centre for Diffraction Data and XRAYAN software [26].

#### 2.3.3. Biomass Estimation

The Bradford protein assay was applied for the determination of the concentration of total protein in the liquid sample [27]. The samples were injected to 96 well plates where the concentrations were determined by the absorbance of the pretreated samples at 595 nm on the Sunrise^TM^ plate reader. The cell counts in liquid samples were analyzed by dye-staining method using 1 mg/L DAPI (6-diamidino-2-phenylindole) solution. After staining, the samples were incubated in the dark for 20 min and subsequently filtered by a 0.2 um black polycarbonate membrane. The membrane was dried for 10 min and then observed on a Nikon Eclipse 80i fluorescence microscope (magnification: 600×; filter set: WU) for the cell counting.

### 2.4. Bioinformatic Analysis and Data Realization

#### 2.4.1. Extraction of DNA and PCR Amplification

The culture was centrifuged, and cell pellet was used for total DNA extraction by FastDNA^®^ SPIN kit. Briefly, the homogenization was carried out in a 2 mL lysing matrix tube through a bead-beating process carried out on FastPre24^®^ (60 s, 6 m/s). The DNA purification includes protein precipitation (4 °C, 10 min), DNA precipitation, and DNA washout. The purified total DNA was set as a template for PCR amplification of the hypervariable V3–V4 regions of the 16S rDNA. The following primers were used: 16S_V3-F: 5′ TCGTCGGCAGCGTCAGATGTGTATAAGAGACAGCCTACGGGNGGCWGCAG 3′ and 16S_V4-R: 5′ GTCTCGTGGGCTCGGAGATGTGTATAAGAGACAGGACTACHVGGGTATCTAATCC 3′. Phusion High-Fidelity DNA Polymerase (Thermo Scientific) was used for PCR reaction, whereas the 16S rDNA fragments were amplified in a thermocycler (Biorad) with 20 cycles. PCR conditions were set up following the description of Dziewit et al. (2015) [28], in short: the KAPA HiFi PCR kit (Roche) was used for PCR amplification. PCR program was set as initial denaturation at 95 °C for 3 min, followed by 26 cycles of 98 °C for 20 s, 63 °C for 15 s and 72 °C for 15 s, and a final extension at 72 °C for 5 min. The total DNA concentration was determined by the NanoDrop 2000 instrument (NanoDrop Technologies). The quality of both total DNA and PCR products were examined by gel electrophoresis.

#### 2.4.2. 16S rDNA Sequencing

The library was prepared by an Illumina TruSeq DNA Sample Preparation Kit adding index adapter for later sample tracing. The 16S rDNA amplicon of the library was then amplified and sequenced on an Illumina MiSeq instrument (Illumina, San Diego, CA, USA) in the DNA Sequencing and Oligonucleotide Synthesis Laboratory of the Institute of Biochemistry and Biophysics, Polish Academy of Sciences. A v3 MiSeq chemistry kit was applied in a paired end mode. After removing low-quality sequences with QC score <25 and ambiguous base N, the remaining high-quality sequences were clustered into an operational taxonomic unit (OTU) at 97% similarity level via UCLUST method. Taxonomy assignment of OTU sequences was performed against Greengenes 13_8 reference. Above analyses were carried out by QIIME 2 [29], whereas the phylogenetic analyses were conducted in MEGA X [30]. OTU sequences were further used to predict metagenome by PICRUSt 2 [31]. PICRUSt normalizes the OTU abundance table and removes the 16S marker gene copy number in each genome. Compare to the COG and KEGG libraries to obtain the KO information corresponding to the OTUs, then calculate the abundance of each COG and KO.

## 3. Results and Discussion

### 3.1. Isolation and Identification of Microbial S[2−] Oxidation Consortium

#### 3.1.1. Structure of the Microbial Community

**Figure 2:**
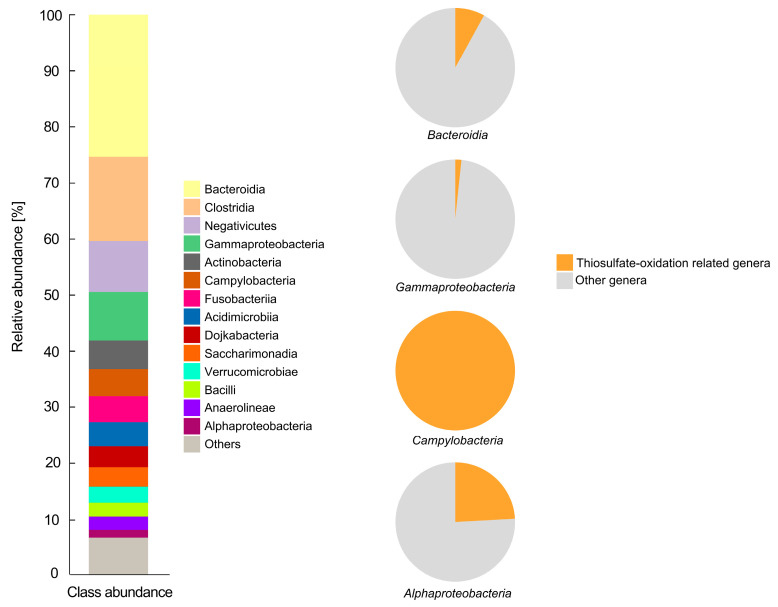
Relative abundance of bacterial classes in the consortium. The abundance at class level was presented in a bar plot. The pie plot shows the proportion of sulfide-oxidizing bacterial (SOB) genera that harbors thiosulfate-oxidation related genes in a specific class (Bacteroidia, Gammaproteobacteria, Campylobacteria, and Alphaproteobacteria, respectively) in which thiosulfate-oxidation related genes, *soxA*, *soxB*, *soxC*, *soxX*, *soxY*, *soxZ*, *sqr*, and *doxD*, were identified.

To understand the genomic sulfide oxidizing capacity of the isolated SOB, the bacterial 16S rRNA genes were analyzed for uncovering community structure (Figure 2). The alpha diversity statistical metrics, measuring diversity within the sample is as follow: Shannon diversity index: 10,445, Pielou’s evenness: 0.948, Faith’s phylogenetic diversity: 38.308. Sequenced sample contains 2070 features with total frequency of 40,233. Mean frequency of a feature was 19.44. Maximum feature frequency was 195 and minimum 1. Our results showed that class Bacteroidia is the dominating group in the consortium (abundance level at 25.36%), followed by Clostridia (abundance level at 8.7%), Gammaproteobacteria (abundance level at 4.93%), and Saccharimonadia (abundance level at 1.40%). The genera potentially harbored thiosulfate-oxidation related genes (*sox*, *sqr*, and *doxD*) were found, including class Bacteroidia, Gammaproteobacteria, Campylobacteria, and Alphaproteobacteria. They account for 8%, 1.67%, 100%, and 24.14% out of each class with 2.03%, 0.15%, 4.93%, and 0.34% relative abundance in the whole community, respectively. It is noted that all Campylobacteria are *Arcobacter*, which is one of the most abundant genera in the consortium (data not shown).

**Figure 3:**
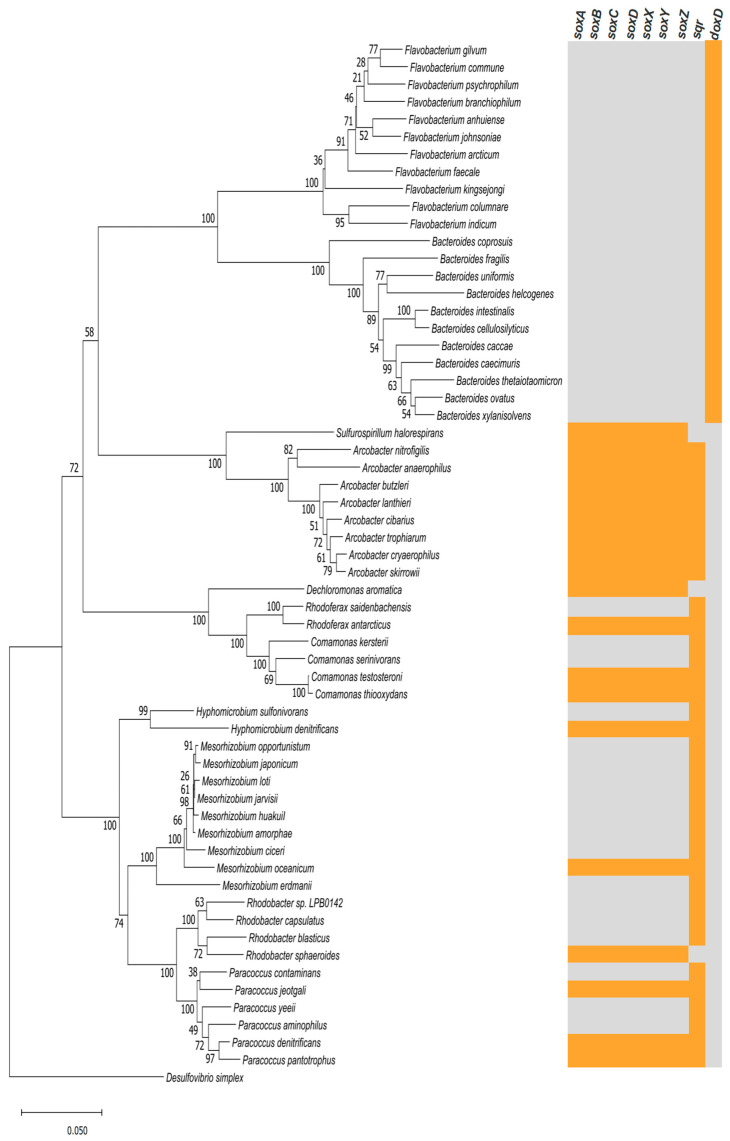
Phylogenetic tree of SOB at species level harboring thiosulfate-oxidation related genes, including *soxA*, *soxB*, *soxC*, *soxX*, *soxY*, *soxZ*, *sqr*, and *doxD*. Orange labeled grids represent the genes positively present, while gray labeled grids represent the genes negatively present. The evolutionary history displayed on tree was inferred using the Neighbor-Joining method. The percentage of replicate trees in which the associated taxa clustered together in the bootstrap test (500 replicates) are shown next to the branches.

A phylogenetic tree of SOB harboring sulfur-oxidization related genes was reconstructed at the species level in Figure 3, and *Desulfovibrio* was set as an outgroup for rooting tree. Eleven species of *Flavobacterium* genus and eleven species of *Bacteroides* genus were found harboring *doxD* gene. This gene was involved in the thiosulfate oxidation process but was not concern with sulfide oxidation. Eight species of *Arcobacter* genus and nine species from genera including *Sulfurospirillum*, *Dechloromonas, Rhodoferax*, *Comamonas*, *Hyphomicrobium*, *Mesorhizobium*, *Rhodobacter*, and *Paracoccus* were identified harboring *sox* genes that mainly functioning in bio-oxidation of inorganic reduced sulfur. Many species, especially those harboring *sox* genes (e.g., Arcobactor), were also found harboring *sqr* genes. These results suggest that the isolated consortium contains a diverse but abundant group of bacteria with sulfur-oxidizing functions. The Arcobacte*r* genus from Campylobacteria class was the main functional group for thiosulfate oxidation in the whole community. This genus was abundantly found in the various environments where sulfide generation occurs [12,13,32,33].

#### 3.1.2. Sulfur Metabolic Pathways Prediction

To reveal the potential roles of microbial consortium in sulfur cycling, we predicted metagenome assigned into KEGG pathway based on 16S rDNA sequence via PIRCUST software [31]. It is noted that this analysis only provides the prediction of the functional gene contents instead of the direct presence of selected genes. We searched the genes in sulfur metabolism, and the results are presented in Table 1. The results show that sulfur metabolic pathways in SOB consortium mainly include sulfur oxidation, sulfur reduction and biosynthesis of organic sulfur. For example, there was a set of genes responsible for the electron binding and transportation in the consortium, which plays an important role in the oxidation of reduced inorganic elemental sulfur or sulfur anions, such as sulfide, polysulfide, thiosulfate, polythionates, and sulfites. For example, they include *soxA* and *soxX* encoding SoxA and SoxX (Cytochrome C proteins) and four genes (*soxB*, *soxC*, *soxY*, and *soxZ*) encoding downstream proteins. The total abundance of *sox* genes is 2.44%. These genes involve a system containing the cytochrome C complex and multienzyme system, which is important for the oxidation of inorganic reduced sulfur [34,35]. Moreover, metagenome prediction also suggested the presence of *doxD* gene (abundance level at 0.38%) encoding thiosulfate dehydrogenase functioning in the thiosulfate oxidation pathway II, and *sqr* gene (abundance level at 1.38%) encoding thiosulfate/sulfide-quinone reductase in the consortium. These results demonstrate that isolated SOB consortium has adequate potentials to oxidize reduced inorganic sulfur, specifically thiosulfate, through different pathways.

Importantly, a considerable abundance of *dsrA* and *dsrB* encoding dissimilatory-type subunit beta that catalyzes the reduction of sulfate/sulfite was showed by prediction, implying the occurrence of spontaneous sulfate reduction in the microbial community. Sulfate-reducing bacteria harboring *dsr* genes use sulfate as electron acceportor producing sulfide [36,37], and this might have a considerable impact on the sulfide oxidation process lowering the efficiency. In addition, a set of genes responsible for other sulfur compound metabolic processes were also adequately predicted in the SOB consortium. The most abundant one is *thiS* gene encoding proteins responsible for the biosynthesis of thiamine diphosphate (abundance level at 4.01%), implying that the SOB consortium possibly produces thiamine (vitamin B1) that stimulates the activity of microbial community as a whole. Other key genes related to sulfur metabolic pathway were also predicted, including *moaD* gene encoding sulfur transporting proteins during molybdopterin biosynthesis, *tau* genes (including *tauA, tauB*, and *tauC*) encoding taurine binding/transporting proteins, and *ssu* genes (including *ssuA*, *ssuB*, *ssuC*, and *ssuD*) encoding alkanesulfonate binding/transporting and oxidation proteins. Notably, our prediction also showed the presence of a *glpE* gene encoding catalytic enzyme towards the reaction from thiosulfate to thiocyanate. These results indicate that SOB are not only for energy conservation and electron transportation through biochemical oxidation and reduction in sulfur cycling but also biosynthesis of organic sulfur that is important for some metabolic activities.

### 3.2. S[2−] Oxidation to Elemental Sulfur—Verification on the Synthetic Media

H_2_S was known to be highly toxic to human and is easily released from the liquid solution through volatilization [38]. For safety reasons and to verify the capability of SOB consortium to oxidize S[2−] to produce elemental sulfur, a semi-continued mode experiment was carried out on synthetic media in the bioreactor, where alkaline Na_2_S solution (instead of H_2_S) was dosing with a constant rate. However, the Na_2_S might still be unstable in the aqueous solution—it produces H_2_S when pH is low, while the oxidation of S[2−] is a proton generation process. We tested controlling the pH at neutral (7–7.5) as proposed by Alcántara et al. (2004) [17], but no significant microbial biomass and sulfur production was observed (data not shown). Although, the alkaline pH might affect microbial composition of the SOB consortium, the slightly basic pH allows for greater dissociation of the sulfide ions for the SOB. Therefore, we kept the pH constantly at ~8.5 to reduce the S[2−] losses and to ensure the adequate metabolic activity for lithoautotrophic SOB.

Figure 1 shows the scheme of the sulfide oxidation system for experiments. The key to the successful biological sulfide oxidation is the O_2_ level and is sometimes represented by S[2−]/O_2_ ratio. Under microaeration conditions (saturation of the solution with oxygen kept at 1%), S[2−] can be efficiently converted to S[0] by SOB [18]. In the case of an O_2_ overdose, S[2−] is chemically or biologically oxidized to sulfate (S[6+]), which generates more heat of reaction [11]. Different S[2−]/O_2_ ratios (from 0.5 to 1.5) were suggested for effective biological sulfide oxidation to produce S[0] (maximum S[0] formation rate reached up to 80%) [16,39]. In this regard, the S[2−] is dosed ~197.8 mg/L/d, whereas the saturation of the solution with oxygen was kept at 1%. Two variants were set up: with and without the consortium. After running for 5 days, we collected the sediments and made the sulfur balance calculation of the process.

#### 3.2.1. Total Sulfur Mass Balance

To illustrate the sulfur cycle during the sulfide oxidation, the total sulfur mass balance was calculated according to the following equations:

M_SI_ ≈ M_SO_ (1)

M_SI_ = I_S[6+]_ × V_a_ + I_S[2−]_ × V_b_ (2)

MSO = (OS[6+] + OS[2−]) × Vc + MS[0](3)

where M_SI_ represents the sulfur amount input (mg); M_SO_ represents the sulfur amount output (mg); I_S[6+]_ is the S[6+] concentration in the inoculum (mg/L); I_S[2−]_ is the S[2−] concentration in the Na_2_S solution dosed to the reactor (mg/L); O_S[6+]_ is the concentration of S[6+] in the reactor after 5 days (mg/L); O_S[2−]_ is the concentration of S[2−] in the reactor after 5 days (mg/L); V_a_ is the volume of the inoculum (L); V_b_ is the volume of the dosed Na_2_S solution within 5 days (L); V_c_ is the total volume of the liquid in the reactor after 5 days (L).

**Figure 4:**
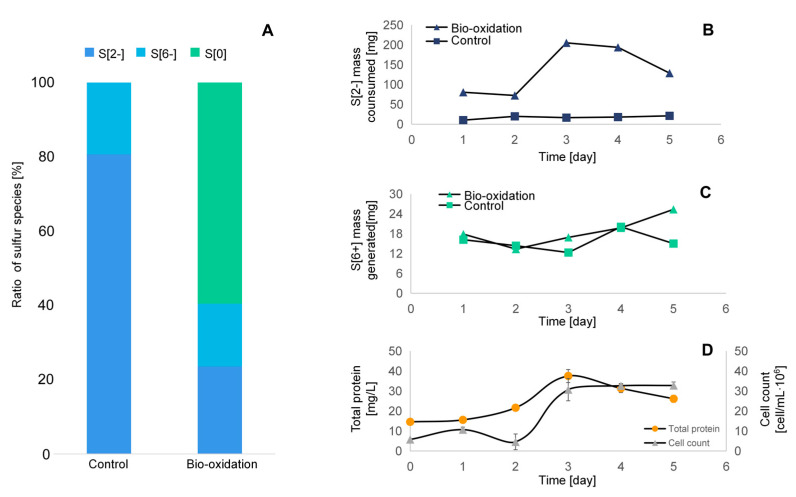
Biological sulfide oxidation on synthetic medium. (**A**) Input and output ratio pattern of sulfur species in the bioreactor (S[2−], S[6+], and S[0]); (**B**) mass of S[2−] consumed each day during 5 days operation; (**C**) mass of S[6+] generated each day during 5 days operation; (**D**) the changes of total protein and cell count in the reactor as a function of time.

The distribution of the sulfur species in the bio-oxidation and control variants is shown in Figure 4A. The input of S[6+] was originated from inoculum and the MB culture where MgSO_4_ was used (here, the S_2_O_3_^2−^ was neglected). In turns, the input S[2−] is from Na_2_S entering the reactor during 5 days by constantly pumping. The input S[6+] was measured and calculated after the inoculation of SOB, which is 124.6 mg, accounting for 12% of the input sulfur. On the other hand, the input S[2−] was calculated as 930.2 mg, accounting for 88% of the input sulfur by knowing the concentration of the Na_2_S stock and the volume dosed to the reactor within 5 days. The output of the sulfur components was verified by measurement and calculation of the S[2−] and S[6+] mass in the liquid culture and the S[0] mass in the collected sediment after 5 days experiment. The collected S[0] (here we assume the collected dry sludge mass equals S[0] mass) accounts for 60% of the total sulfur after bio-oxidation; in contrast, no S[0] was obtained in the control variant. The S[6+] accounts for 19% and 17% of the total sulfur of output for bio-oxidation and control variant, respectively. This indicates that there was no significant biological sulfate reduction in the culture, because the adopted medium contains no carbon and energy source for sulfate-reducing bacteria. A significant difference regarding the proportion of S[2−] to the total sulfur output was observed on bio-oxidation (81%) and control variant(24%). Here, we did not consider the deviation caused by equipment and operation, as well as sulfide losses caused by volatilization and production of other sulfur compounds, such as thiosulfate (see Section 3.2.4). This leads to a gap in the calculation of the full balance of sulfur. Nevertheless, these results indicated that with the same dosage of S[2−], S[0] was effectively formed by bio-oxidation of S[2−], whereas in the control variant, only chemical oxidation took place, which leads to negligible S[6+] generation. Within 5 days of operation, the transformation rate from S[2−] to S[0] by SOB reached ~84%.

#### 3.2.2. Sulfur Species as a Function of Time

For a simple presentation of the dynamic influence of SOB on the sulfur oxidation state changes, the S[2−] consumed and S[6+] generated each day was calculated from the following equations:

M_S[2−]n_ = (V_0_ + n × V_d_) × O_S[2−]n_ (4)

M_S[6+]n_ = (V_0_ + n × V_d_) × O_S[6+]n_ (5)

CM_S[2−]n_ = C_S[2−]_ × V_d_ + M_S[2−]n_ − M_S[2−](n+1)_ (6)

CM_S[6+]n_ = M_S[6+]n_ − M_S[6+](n+1)_ (7)

where CM_S[2−]n_ is the consumed S[2−] mass at day n (mg); CM_S[6+]n_ is the generated S[6+] mass at day n (mg); M_S[2−]n_ is the S[2−] in the reactor at day n (mg); M_S[6+]n_ is the S[6+] in the reactor at day n (mg); M_S[2−](n+1)_ is the S[2−] in the reactor at day n+1 (mg); M_S[6+](n+1)_ is the S[6+] in the reactor at day n+1 (mg); C_S[2−]_ is the concentration of S[2−] in the Na_2_S solution dosed to the reactor, equals 4000 (mg/L); O_S[2−]n_ is the concentration of S[2−] at day n; O_S[6+]n_ is the concentration of S[6+] at day n; V_d_ is the volume of the liquid pumped to the reactor each day (L); V_0_ is the volume of the liquid in the reactor at day 0.

Figure 4B,C shows the amount of the S[2−] consumption and S[6+] generation for bio-oxidation and control as a function of time, respectively. The S[6+] generation rate at each day for both two variants shows flat changes, generally going up and down from 10.2–21.1 g (control) and 12.3–20.1 g (bio-oxidation), respectively. This indicates that very limited oxidation of S[2−] to S[6+] appeared in both variants. It is worthy to mention that the bio-oxidation did not affect the S[6+] generation from S[2−] on a significant level, compared to the control, which implies effective S[0] production by the SOB thanks to the low oxygen concentration supplied. The S[6+] generation amount on the two curves roughly coincides with each other, except for the last day where a slightly higher amount of S[6+] is produced. The culture was fed continuously at a constant rate in S[2−]. In the first 2 days of bio-oxidation, the S[2−] consumption rate was low, and only 72.4–80.5 g of S[2−] was consumed per day, which is likely due to the adaption phase of SOB. Only after three days, the oxidation efficiency was dramatically increased where 205 g of S[2−] was consumed, while the S[6+] concentration remains stable, indicating the highest sulfide oxidation appearing. It was proved that the SOB is capable to produce S[0] fairly fast [39]. After that time, the S[2−] consumption efficiency started to decrease, ending up with 129.7 g/day. Unoxidized sulfide accumulated in the reactor and might be responsible for the decrease of sulfide consumption. This is probably because the chemical oxidation of S[2−] is restricted when bio-oxidation is applicable. This is in contradiction to the results reported by another research that is the substances in the inoculum certainly catalyze the chemical sulfide oxidation [40]. In the control variant, an almost unchanged amount of S[2−] was consumed each day, which resulted from either chemical oxidation or the physical losses of S[2−] (volatilization of H_2_S).

#### 3.2.3. Growth Curve of SOB during Sulfide Bio-Oxidation

The location of sulfide oxidation by SOB was reported to be either in the biofilm sticking on the surface of the reactor or in the liquid culture [11,32,41]. A very thin biofilm layer was found on the surface of the culture, which is likely due to the frequently immediate contact of SOB with dosed S[2−] substrate, attracting sulfide oxidizers. However, we did not observe the formation of biofilm on the wall of the bioreactor nor the mixer. In contrast, abundant bio-activity was detected (total protein from 14.56 to 37.51 mg/L) in the culture liquid, indicating that the sulfide oxidation occurred mainly in suspended cells. A previous study reported that the liquid-based processes have better performance compared to biofilm-based processes in terms of sulfide oxidation [32].

The changes in total protein and cell count of the SOB as a function of time were measured, and the results were presented in Figure 4D. During the 5 days experiment, the total protein concentration ranged from 14.6 to 26.1 mg/L, whereas the number of cells ranged from 4.6 to 32.75∙10^9^ cell/L. The total protein slowly increased in the first two days from 14.6 to 15.6 mg/L. It sharply raised from day 2 to day 3 to up to 37.5 mg/L and slightly dropped thereafter. In terms of the cell count, even with dynamic feeding of the sulfide, which is the energy source of SOB, a clear lag phase from day 0 to day 2, and a logarithm phase from day 2 to day 4 were observed. Both the highest concentration of the total protein and cell counts were observed on the third day, which corresponds to the results of the S[2−] consumption efficiency of bio-oxidation. The growth yield in the first three days reached ~2.34 g_protein_/mol_sulfid_ by calculation for the SOB consortium, which is similar to a previous study (2.85 g_protein_/mol_sulfid_) [16]. Other studies reported growth yield at 3.1 to 5.8 g_biomass_/mol_sulfid_ of single strains in aerobic batch cultures [42,43].

#### 3.2.4. XRD Analysis of Sediments

**Figure 5:**
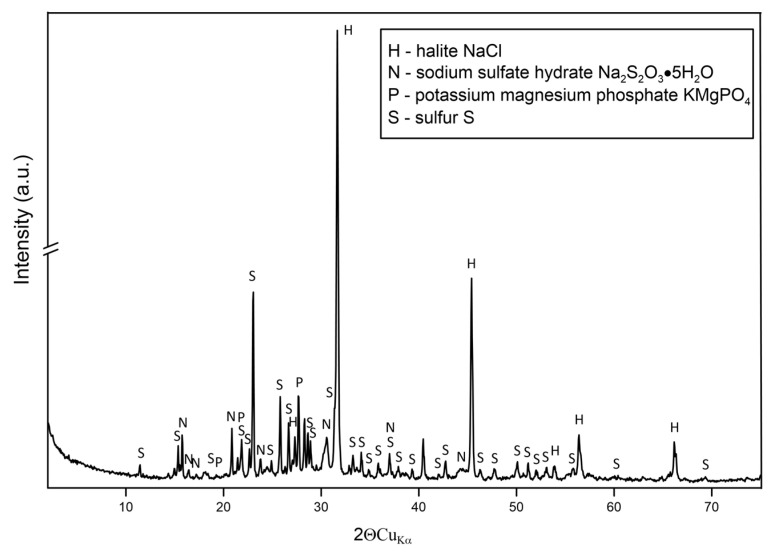
X-Ray Diffraction pattern of the collected sediment from the reactor after 5 days of operation.

The collected sulfur containing sediments has no specific stratification, which is in line with Kobayashi et al. (2012) [32], but contrary to Ramos et al. (2014) [41]. The X-ray Diffusion analysis revealed the sulfur composition in the collected sediments (Figure 5). The S[0] was the dominant species of sulfur found in the sediment. Meanwhile, Na_2_S_2_O_3_·5H_2_O was also detected. The formation of thiosulfate is presumably due to either chemical or biological oxidation of S[2−] [39,44]. Especially when the oxygen is not used fast enough by SOB, the chemical oxidation to thiosulfate becomes obvious. It has been reported that the presence of products such as thiosulfate and polysulfide is a clear indication of a decrease of SOB activity [17]. NaCl was found which originated from the huge consumption of HCl supported by the pH balance system to keep the constant pH at 8.35. The presence of KMgPO_4_ in the sediment can result from the reaction of potassium phosphate and magnesium sulfate from the modified MB medium, under the alkaline pH condition.

To summarize, the isolated SOB can produce S[0] by oxidizing S[2−] in a synthetic medium. A ~84% conversion rate was obtained when the pH (8.35) and oxygen level (1%) was controlled in a semi-continuous bioreactor after 5 days of operation. The highest growth rate (31.35 mg/L) and S[2−] oxidation rate (204.97 mg/day) were both reached at the third day, implying an adequate adaption time is required for bacterial metabolism. The sulfate concentration remains stable during the whole process. This indicates that limiting oxygen supply successfully prevented the complete oxidation of S[2−] (to S[6+]). We made correlation analysis (Pearson) between sulfide mass and biomass in the medium and obtained significant correlations (*p* < 0.01 and *p* < 0.05 for cell count and total protein, respectively). Thiosulfate is formed besides S[0], however, with a low proportion presenting in the collected sediment. Our results of S[0] bio-conversion are comparable to a few studies [16,45] and are better than some other studies [39,46,47]. Although we proved that our SOB is effective in transforming S[2−] to S[0], in real industrial effluent, many chemical-physical conditions hinder the activity of isolated bacteria consortium dedicated for specific purposes [48]). It is therefore essential to verify the activity/efficiency of isolated SOB in effluents, specifically, after biological sulfate reduction from hydrometallurgical waste leachate that requires sulfide depletion.

### 3.3. Bioaugmented Treatment of the S[2−] Containing Industrial Effluent

Controlled oxygen supply (also known as micro aeration) is the key point for effective sulfide oxidation producing S[0] and avoiding complete oxidation to S[6+] [39,49]. However, low oxygen does not fit the requirement regarding the removal of organic matters, which is often associated with S[2−] in most of the industrial effluents [50,51]. Because in microaerobic condition, the decomposition of organic matters requires a fairly long operation time and huge space, which often stands in the way of opportunities for industrial wastewater treatment. Therefore, we adopted a two-stage oxidation for the treatment of such effluent. At the first stage, the isolated SOB consortium was introduced to the effluent under microaerobic condition (oxygen saturation at 1%). While after 5 days of sulfide oxidation, the saturation of the solution with oxygen was boosted up to 100% and a commercial BioRemOil consortium (http://rdls.pl/, accessed on 1 December 2020) was added to remove COD. The main advantages of employing such two-stage bio-oxidation are that the excessive S[2−] and COD are removed in situ separately where additional end-of-pipe units were reduced. The main drawback is the additional residence time required to deposit the produced elemental sulfur. In our case, we do not need this process because the amount of produced S[0] is too low. However, this should be well considered when the proposed technology is applied in a rich-sulfide condition.

#### 3.3.1. Characterization of the Industrial Effluent

Industrial effluent was obtained after biological sulfate reduction: In general, spent alkaline batteries were leached by sulfuric acid, resulting in the acidic leachate that requires treatment for sulfate removal and metal recovery (Fe, Mn, and Zn). In this scenario, biological sulfate reduction with neutralization approaches was employed, producing sulfide ions precipitating metals. However, an inevitable problem is the high concentration of sulfide, which affects microbial activities and can be released to the environment. Another problem is the high amount of COD, including VFA generated during the anaerobic sulfate reduction process, boosted by technical solutions (e.g., acetate and ethanol) [52]. The detailed chemical characterization of the effluent used in this study was carried out, and Table 2 shows the results. The sulfate concentration is ~331.7 mg/L, and is under the standard of polish legislation (500 mg/L) (Regulation by the Minister of Environment of Poland, 2014). The pH is also close to neutral. However, the sulfide and COD found in the effluent is ~75.3 and 4703.3 mg/L, which would need further management.

#### 3.3.2. Two-Stage Oxidation of the Industrial Effluent

**Figure 6:**
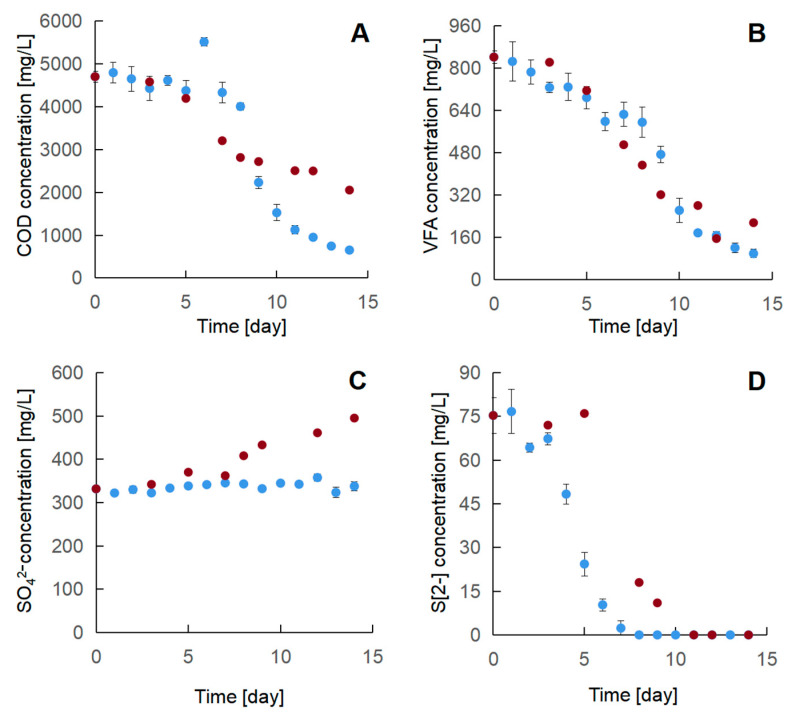
Changes in chemical parameters as a function of time within 14 days of two-stage bio-oxidation and control experiment: (**A**) COD; (**B**) VFAs; (**C**) SO_4_^2−^; (**D**) S^2−^. Blue dots refer to data of bio-oxidation using SOB and Bioremoil. Red dots refer control without adding microbial consortia (saturation of the solution with oxygen was kept at 1%from day 0 to 5), then, oxygen level was increased and saturation of the solution with oxygen was kept at 100% from day 6 to 14. Day 0—Inoculation of SOB; day 6—Inoculation of BioRemOil.

To realize the effectiveness of the proposed two-stage oxidation strategy, we measured the concentration of COD, VFA, SO_4_^2−^, and S[2−] throughout the process. Figure 6 shows the results as a function of time. In general, the sulfide oxidation was realized at the first stage by the application of SOB at a microaerobic condition with an oxygen saturation level at 1%. The COD and VFA were mainly removed at the second stage with the application of BioRemOil, where oxygen saturation level was adjusted to 100%. A similar trend of S[2−] concentration changes was found in the control variant. However, the sulfate concentration increased. In the bio-oxidation batch, the S[2−] concentration was significantly decreased to 24.3 mg/L in the first stage (day 0–5), with an initial concentration at 75.3 mg/L. From day 6 to the end of the experiment, the residual S[2−] was almost completely removed at a high oxygen level in the culture. No significant amount of S[0] sediment was obtained. Regardless, the sulfate concentration was stable, fluctuating from 322.0 to 357.7 mg/L during the whole process. In contrast, the S[2−] concentration remains stable in the first stage of the control variant, while decreased in the second stage, where the sulfate concentration increased. The loss of S[2−] approximately equals to the S[6+] generation from sulfate, implying that no S[0] was produced. These results indicate that a sulfur production was successfully achieved by implementing SOB with the use of the two-stage aeration strategy.

According to the results of bio-oxidation, the concentration of COD was stable from day 0 to day 5, from 4376.7 to 4796.7 mg/L. It is known that when the oxygen dosing rate is low at a different COD load in the effluent, the complex organic substrates that are oxidized cannot be significant [18]. At day 6, an odd peak appeared in the curve, significantly exceeding the initial COD concentration, which was caused by the nutrients in the BioRemOil preparation. From day 6 to day 12, the COD was efficiently and quickly removed from 5516.7 to 952.0 mg/L. After that time, the COD concentration changed slowly, ending up with almost 86.1% of the depletion rate. A similar trend was observed in the case of the VFA changes over time. VFA (13.5%) was removed from day 0 to 5, whereas 59.4% was removed from day 6 to 14. In the first 5 days, the VFA concentration moderately decreased. The presence of oxygen (even at microaerobic level) entails possible partial oxidation of organic substrates. This is likely due to the indigenous microorganisms which utilize these low molecular weight compounds. The sulfate-reducing bacteria might be one of the functional microbes as the effluent provides them the perfect electron acceptor (sulfate) and carbon source (VFA). However, the sulfate concentration was stable, while the sulfide concentration decreased. This indicates that complete sulfide oxidation to sulfate partially took place in the first stage, which approximately balanced the sulfate consumption by microbial sulfate reduction. This is confirmed by the result of the control variant, that is, the sulfate concentration slightly increased in the first stage. At the second stage, VFA concentration decreased rapidly, ending at 98.3 mg/L. In the control variant, the COD and VFA concentration changes as a function of time were similar to that in the bio-oxidation. In the first 5 days, very limited COD and VFA removal was realized. At day 6, the COD and VFA removal markedly raised, followed by the increase of oxygen concentration from 1% to 100%. The final concentration of COD and VFA after 14 days reached 2054 and 215 mg/L, respectively, indicating that oxygen strikingly improves the activity of indigenous bacteria, decomposing the organic substrates in the effluent. These results suggest that most of the organic substrates, especially VFA, in the effluent can be simply removed by increasing the oxygen concentration. On this basis, the application of the bacteria consortium considerably improves their removal efficiency.

## 4. Conclusions

In conclusion, a SOB consortium that can produce elemental sulfur by microbial sulfide oxidation was isolated from post-mining wastes. Metagenome analysis of the SOB showed that *Acrobacter* may be the main functioning genus responsible for sulfur production, because it contains a complete set of genes encoding sulfide-oxidizing system and is the most represented among the consortium. The activity of the SOB consortium was confirmed in both synthetic medium and industrial effluents. However, to manage industrial effluents after sulfate reduction, the depletion of both sulfide and COD is needed. By controlling the oxygen supply, we developed a two-stage strategy using the SOB consortium and another commercial bacterial consortium, whereas fast removal of sulfide and COD (including VFA) were achieved. Our work presents a sustainable model for aqueous sulfide depletion in industrial effluent containing significant organic matter. However, few questions are still unclear with our concerns: (i) how to separate/collect the produced sulfur from the industrial effluent; (ii) what changes in the microbial community take place after sulfur production, and (iii) is it possible to effectively adopt SOB in a passive system with lower costs and lower energy demand.

## Figures and Tables

**Table 1:** The abundance of genes in sulfur related pathways predicted by PIRCUST software based on 16S rRNA abundance. The metabolic pathways were predicted using KEGG Orthology database.

KO Number	Gene	Encoding Protein	Abundance (%)
K02439	*glpE*	thiosulfate sulfurtransferase	0.31
K03154	*thiS*	sulfur carrier protein for thiamime biosynthesis	4.01
K03636	*moaD*	molybdopterin synthase sulfur carrier subunit	2.25
K04091	*ssuD*	alkanesulfonate monooxygenase	1.50
K10831	*tauB*	taurine transport system ATP-binding protein	0.30
K15551	*tauA*	taurine transport system substrate-binding protein	0.30
K15552	*tauC*	taurine transport system permease protein	0.37
K15553	*ssuA*	sulfonate transport system substrate-binding protein	1.68
K15554	*ssuC*	sulfonate transport system permease protein	1.14
K15555	*ssuB*	sulfonate transport system ATP-binding protein	1.47
K16937	*doxD*	thiosulfate dehydrogenase (quinone) large subunit	0.38
K16950	*asrA*	sulfite reductase subunit A	0.4
K16951	*asrB*	sulfite reductase subunit B	0.36
K17218	*sqr*	sulfide (quinone) oxidoreductase	1.38
K17222	*soxA*	sulfur-oxidizing protein SoxA	0.34
K17223	*soxX*	sulfur-oxidizing protein SoxX	0.34
K17224	*soxB*	sulfur-oxidizing protein SoxB	0.29
K17225	*soxC*	sulfane dehydrogenase subunit SoxC	0.31
K17226	*soxY*	sulfur-oxidizing protein SoxY	0.72
K17227	*soxZ*	sulfur-oxidizing protein SoxZ	0.44

**Table 2:** Chemical characteristics of the industrial effluent.

Parameter	Measured Value
COD [mg/L]	4703.3 ± 128.6
VFA [mg/L]	911.7 ± 12.6
NH_4_-N [mg/L]	180.0 ± 2.0
T-N [mg/L]	246.0 ± 3.6
NO_3_^−^-N [mg/L]	49.0 ± 1.7
SO_4_^2−^ [mg/L]	331.7 ± 1.5
S[2−] [mg/L]	75.3 ± 6.1
pH	7.2 ± 0.3

## Data Availability

Sequencing data generated within this study are deposited in NCBI Genbank under the BioProject ID PRJNA705003.
